# Geometric morphometrics of different malocclusions in lateral skull radiographs

**DOI:** 10.1007/s00056-016-0057-x

**Published:** 2016-10-31

**Authors:** Josef Freudenthaler, Aleš Čelar, Christopher Ritt, Philipp Mitteröcker

**Affiliations:** 1 Medical University of Vienna, Vienna, Austria; 2 School of Dentistry, Orthodontics, Sensengasse 2a, 1090 Vienna, Austria; 3 Zahnarzt, Marktplatz 15, 3352 St. Peter in der Au, Austria; 4 Universität Wien, lnstitut für Anthropologie, Vienna, Austria

**Keywords:** Craniofacial shape variation, Shape variation, Facial growth, Open bite, Variation kraniofazialer Form, Formvariation, Gesichtswachstum, Offener Biss

## Abstract

**Background:**

To evaluate the role of craniofacial shape in malocclusion by application of geometric morphometrics to a set of two-dimensional landmarks and semilandmarks obtained from lateral skull radiographs.

**Methods:**

Cephalometric radiograph tracings of 88 untreated Caucasians (age range 7–39 years) were assigned to four groups according to their occlusion: neutrocclusion, distocclusion, mesiocclusion, and anterior open bite. The geometric morphometric shape analysis incorporated 66 landmarks and semilandmarks, which underwent generalized Procrustes analysis, between-groups principal component analysis, thin-plate spline deformation grid visualization, permutation tests, and receiver operating characteristic curves.

**Results:**

The position and shape of the mandible contributed to differences between the distocclusion and mesiocclusion groups, whereas the maxillary shape showed less variation. The growth-related shape alteration during adolescence was most pronounced in the mesiocclusion group and least pronounced in the neutrocclusion group. The open bite group was associated with an altered orientation of the mandibular body and the maxilla,  showed the most hyperdivergent maxillomandibular pattern but was not an own skeletal entity. Despite clear differences in mean shape across the four groups, the individual distribution of craniofacial shape overlapped between the groups without discrete clusters.

**Conclusions:**

Craniofacial shape was clearly associated with dental malocclusion and showed considerable variation. Geometric morphometrics was a powerful research tool but for diagnosing individual malocclusion standard cephalometric measurements including overjet and overbite were equally or more efficient than geometric morphometric descriptors.

## Zusammenfassung

**Hintergrund:**

Untersuchung der kraniofazialen Formvariation und ihrer Assoziation mit 4 Okklusionstypen mittels geometrisch-morphometrischer Analysen zweidimensionaler Messpunkte auf seitlichen Schädelröntgenbildern.

**Methodik:**

Untersucht wurden Durchzeichnungen der Fernröntgenseitenbilder von 88 unbehandelten Europäern im Alter zwischen 7 und 39 Jahren, die anhand ihrer Verzahnung 4 Gruppen zugeteilt worden waren: Neutralokklusion, Distalokklusion, Mesialokklusion und anterior offener Biss. Die geometrische Morphometrie bezog 66 (Semi-)Landmarken ein, diese wurden einer Prokrustesanalyse und einer “between-groups principal component analysis” unterzogen, in einem Thin-Plate-Spline-Deformationsgitter visualisiert und mit Permutationstests und ROC (“receiver operating characteristic”)-Kurven evaluiert.

**Ergebnisse:**

Position und Gestalt des Unterkiefers trugen zu den primären Unterschieden zwischen der Distal- und Mesialokklusionsgruppe bei. Die Form der Maxilla dagegen zeigte deutlich weniger Variation. Wachstumsbedingte Formveränderungen während der Adoleszenz waren in der Mesialokklusionsgruppe am stärksten, in der Neutralokklusionsgruppe am wenigsten ausgeprägt. In der Gruppe mit anterior offenem Biss fiel eine Umorientierung des Corpus mandibulae und der Maxilla bei maximal hyperdivergentem Muster auf, doch stellte diese Gruppe keine eigene skelettale Entität dar. Trotz klarer Unterschiede zwischen den morphometrischen Durchschnittsformen der 4 Okklusionsgruppen überlappten die individuellen Formkonfigurationen zwischen den Gruppen, ohne dass Cluster beobachtet wurden.

**Schlussfolgerung:**

In der vorliegenden Studie bestanden eindeutige Verknüpfungen zwischen kraniofazialer Form und Okklusionstyp, sie unterlagen aber erheblicher Variation. Die geometrische Morphometrie hat sich als effektive Forschungsmethode bewährt. Für die individuelle Diagnose einer Malokklusion waren jedoch kephalometrische Standardmessungen einschließlich Overjet und Overbite gleich effizient oder sogar effizienter als geometrisch-morphometrische Deskriptoren.

## Introduction

Thorough diagnosis and treatment planning of malocclusions require a distinct understanding of both dentoalveolar and skeletal components in anterior–posterior and vertical directions [17, 33, 34]. While Angle’s classification is based on dental criteria, lateral cephalometric headfilms display the skeletal framework in association with the dentition [30, 36].

Conventional radiograph cephalometry combines linear and angular measurements or indices derived from these measurements [18, 35]. For research, however, cephalometric measurements may not suffice a detailed description of the craniofacial morphology [5, 13, 15, 25, 29]. These shortcomings can be overcome by geometric morphometrics (GM), a method based upon the Cartesian coordinates of landmarks. After standardizing landmark configurations for overall position, scale, and orientation, the resulting shape coordinates represent the shape of the configurations only, whereas the overall size of the configurations is explicitly represented by a single variable: centroid size, a standard measure of the overall size of a landmark configuration. Centroid size equals the square root of the summed squared distances of the landmarks from their centroid (average landmark position).

Because of the powerful visualization techniques and the typically large number of variables, GM allows for effective exploratory studies and statistical tests [5, 25]. GM has been applied in orthodontic research to study growth, treatment effects, and shape variation [6–8, 12, 15, 16, 20, 21].

In the present study we applied GM including between-groups principal component analysis (bgPCA) in order to (1) compare craniofacial shape across the Angle classes and open bite malocclusion in a sample of juvenile and adult untreated patients. (2) We investigated if different types of occlusion were associated with a particular skeletal pattern. (3) We assessed individual variation within the malocclusion groups and quantified (4) to which degree the diagnosis of malocclusion can be inferred from skeletal morphology itself. For this purpose we contrasted the performance of GM descriptors with classic measurements.

## Materials and methods

The sample comprised 88 Caucasians aged from 7 to 39 years (median 12 years; details shown in Table 1). In all, 28 males and 60 females were pooled together for all analyses. Inclusion criteria embraced complete mixed and permanent dentitions without consideration of third molars. Exclusion criteria were dental agenesis except third molars, cleft palate, craniofacial syndrome, former orthodontic treatment, and prosthetic therapy. Following the assignment protocol of Ellis and McNamara [9] and Singh et al. [35], pretreatment study casts of the patients were used to diagnose either an Angle class I (neutrocclusion), class II (distocclusion), class III (mesiocclusion), or open bite malocclusion (zero or negative overbite). The institutional ethics committee approved this investigation (no. 1033/2012).

**Tab. 1:** Number of patients and distribution of age in years for the diagnostic groups **Tab. 1** Anzahl der Patienten und Altersverteilung in den diagnostischen Gruppen

	Class I	Class II	Class III	Openbite
Females	18	25	8	9
Males	9	13	3	3
Total	27	38	11	12
Min/max age	7/30	7/33	8/18	7/39
Median age	11	12	13	11.5

The lateral cephalometric radiographs originated from two private practices, a public health care institution, and the dental university, all situated in Vienna, Austria. The radiographs had been taken for orthodontic treatment planning. Two authors traced the X-rays on matte acetate sheets. Intra- and interoperator reliabilities of the tracings were tested with Dahlberg’s equation yielding errors ranging from 0.3 to 1.3 mm (mean 0.7 mm) and 0.5°–1.8° (mean 1°). A third investigator, who was blind to the diagnosis, made scans of the manual tracings and digitized the following 16 anatomical landmarks using the TPSdig 2.0 software (James Rohlf, State University of New York, Stony Brook, NY, USA): nasion, orbitale, sella, the most superior point on the clivus, basion, the highest point on the coronoid process, condylion, anterior nasal spine, posterior nasal spine, supradentale, infradentale, the intercuspal indentions of the averaged maxillary and mandibular first molars, superior incision, inferior incision, and the apices of the maxillary and mandibular incisors.

**Fig. 1:**
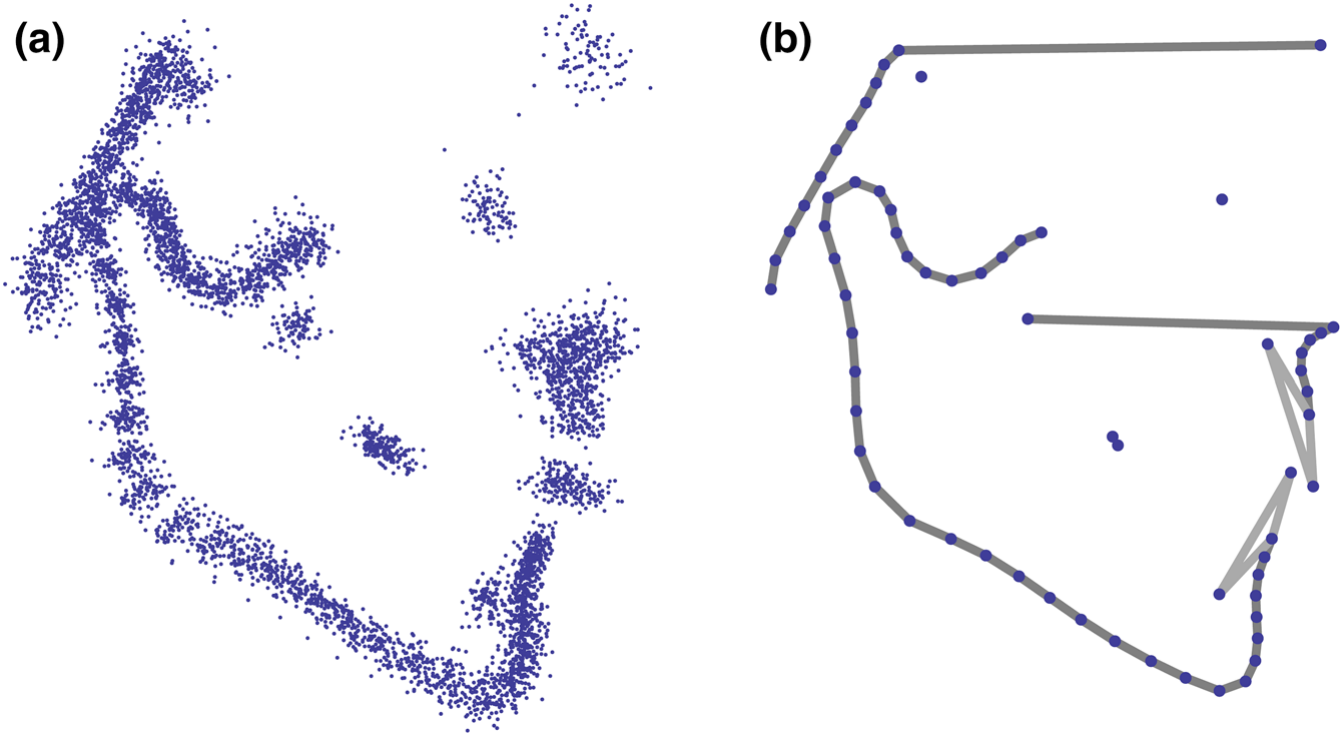
**a** Scatterplot of the superimposed landmark configurations (Procrustes shape coordinates). **b** Visualization of the sample mean shape **Abb. 1 a** Scatter-Plot der überlagerten Landmarken-Konfigurationen (Prokrustes-Koordinaten). **b** Visualierung der Durchschnittsform in der Patientengruppe

In addition, the outlines of the mandible, the clivus, and the maxilla between posterior nasal spine and prosthion were measured by a total of 50 semilandmarks, i.e., points on smooth curves, for which the exact location on the curve cannot be identified and hence is statistically estimated. We used the sliding landmark algorithm for this purpose, which minimized the bending energy, a measure of local shape difference, between each individual and the sample average [5, 23]. This approach allowed for the joint analysis of homologous points (anatomical landmarks) and curves represented by semilandmarks (Fig. 1; Table 2).

**Tab. 2:** Group means and standard deviations for selected angular and linear measurements **Tab. 2** Durchschnittswerte und Standardabweichungen für ausgewählte Winkel- und lineare Messungen

	Gonial angle	PP–MP	N-S-Ba	SN–PP	SN–MP	U1-SN	L1-MP	Maxillary length	Distance S-N
Class I	129.5 (7.4)	27.5 (5.9)	128.5 (5.0)	6.8 (3.4)	34.1 (7.2)	101.8 (5.2)	90.0 (5.2)	53.5 (3.6)	71.3 (3.8)
Class II	126.7 (6.3)	26.7 (5.3)	130.2 (5.4)	5.7 (3.2)	32.3 (5.8)	103.6 (10.0)	94.0 (10.0)	54.4 (3.9)	71.3 (4.3)
Class III	131.8 (5.1)	30.6 (4.3)	132.3 (5.1)	7.6 (3.0)	38.2 (6.6)	103.3 (5.8)	82.5 (5.8)	52.8 (3.3)	69.2 (5.0)
Openbite	130.1 (5.3)	31.7 (6.6)	130 (8.4)	6.5 (3.8)	36.7 (7.2)	105.5 (8.9)	90.0 (8.9)	55.1 (4.4)	69.2 (4.9)

Gonial angle, angle between palatal plane and mandibular plane (PP–MP), cranial base angle (nasion-sella-basion, N-S-Ba), angle between sella-nasion line and palatal plane (SN–PP), angle between sella-nasion and mandibular plane (SN–MP), inclination of maxillary incisor to sella-nasion line (U1-SN), inclination of mandibular incisor to mandibular plane (L1-MP), maxillary length in millimetres (distance between anterior and posterior nasal spines), distance sella-nasion (S-N) in millimetres

After sliding the semilandmarks, all 88 landmark configurations were superimposed by Generalized Procrustes Analysis, a least-squares oriented registration technique, standardizing position, scale, and orientation of all configurations [31]. Procrustes registration does not require specification of a reference plane because it is based on all landmarks and semilandmarks [15]. The resulting Procrustes shape coordinates contain only information on the shape of the landmark configurations and are the basis for further statistical analysis.

We computed and visualized the average shape of all four diagnostic groups, regardless of the age. In addition, we linearly regressed the shape coordinates on age to model age-dependent shape variation within each of the four groups. Gender was used as a covariate in these regressions to account for the heterogeneity of the age distribution between the sexes. The multivariate pattern of individual and group mean differences was assessed by bgPCA [24], an ordination technique in-between the more familiar approaches of principal component analysis and discriminant function analysis. It leads to a low-dimensional representation of the high-dimensional shape space that maximizes the variation between the group means. Thin-plate spline deformation grids were used to visualize average shape differences between diagnostic groups and age effects.

The statistical significance of group mean differences was assessed by permutation tests, using 5000 random permutations and the Procrustes distance between the group mean shapes as the test statistic. Permutation tests do not require normally distributed variables and can be applied to multivariate datasets that are not of full rank, such as Procrustes shape coordinates.

To investigate whether the patients can be successfully classified to their diagnostic groups when using only bony structures, without reference to the teeth, we computed leave-one-out classifications based on multiple-group linear discriminant functions for two different datasets, one containing only skeletal landmarks and another one containing only dental landmarks (molars and incisors). We used the first five ordinary principal components (PCs) of each dataset for the classification. A higher number of PCs did not increase classification success. In addition, we computed receiver operating characteristic (ROC) curves [39] based on the bgPCs for (1) the full dataset, (2) the skeletal landmarks, and (3) the linear measurements overjet and overbite. The ROC curve plots the true positive rate (sensitivity) against the false-positive rate (1—specificity).

## Results

**Fig. 2:**
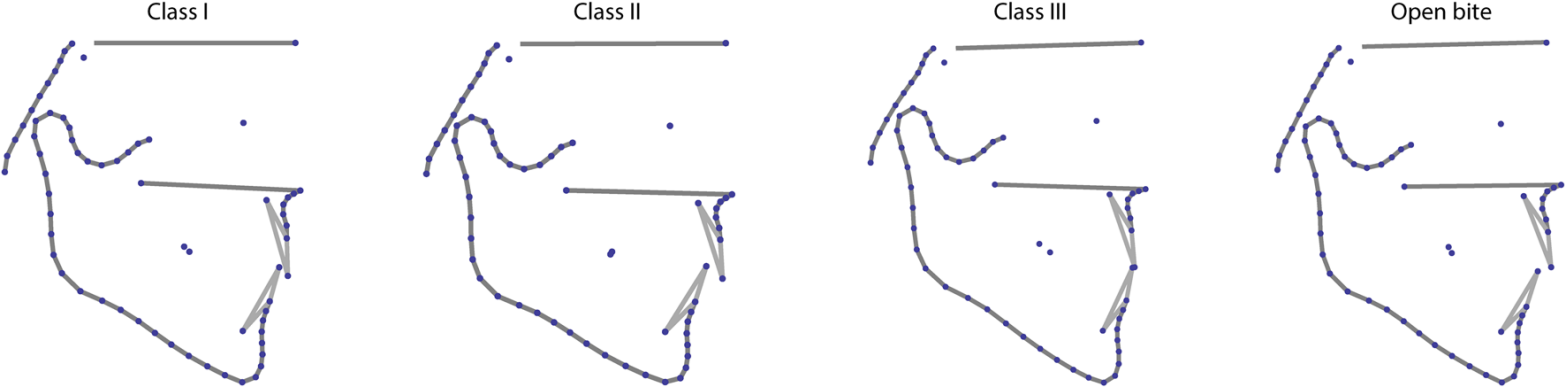
Mean shapes of the four diagnostic groups **Abb. 2** Durchschnittsformen der 4 diagnostischen Gruppen

**Fig. 3:**
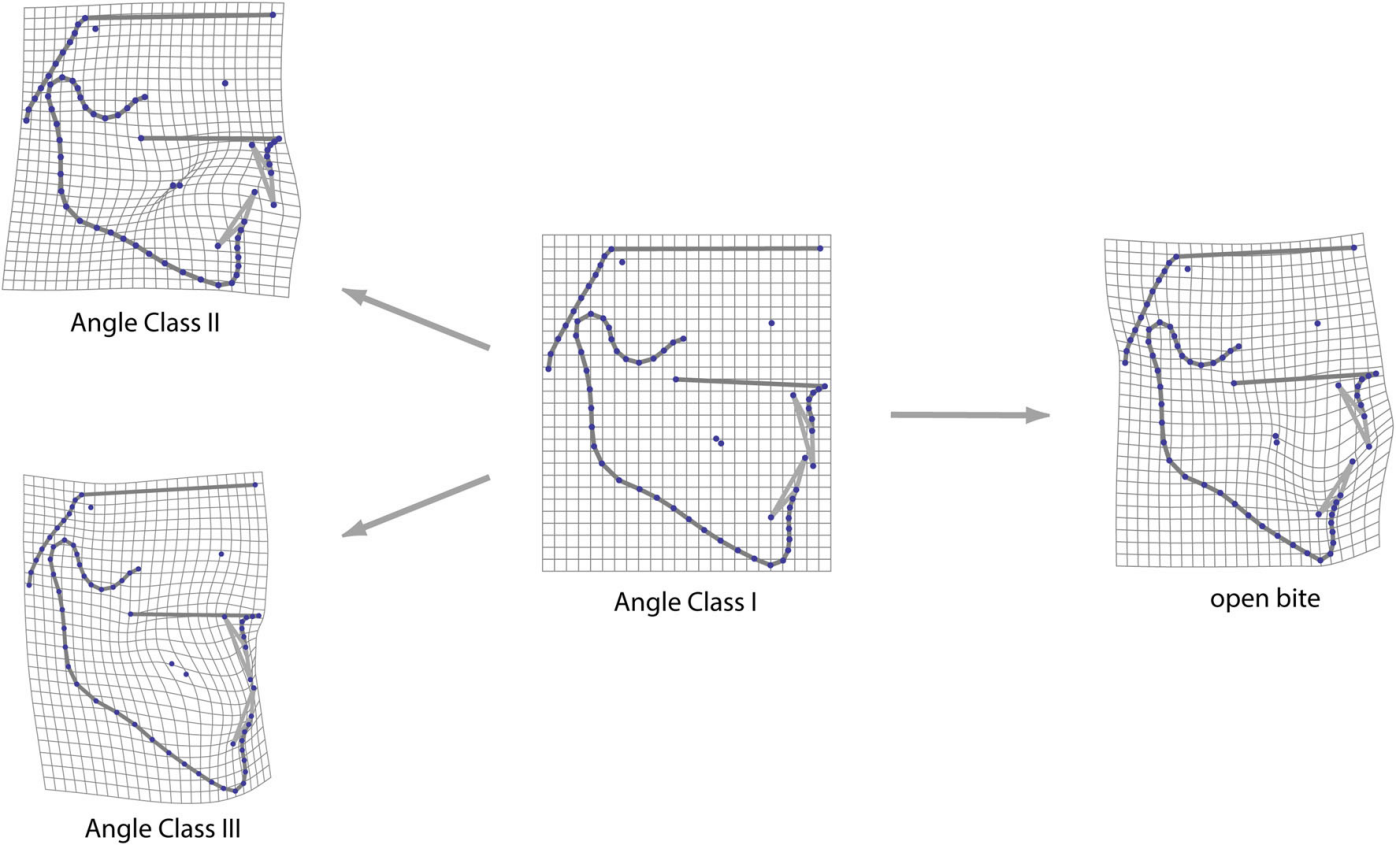
Deformation grids illustrating the mean shape differences between Angle Class I group and the three types of malocclusion. Shape differences are linearly extrapolated by factor 2 **Abb. 3** Deformationsgitter zur Darstellung der Formunterschiede zwischen der Klasse-I-Gruppe und den 3 Arten der Malokklusion. Formunterschiede sind linear mit dem Faktor 2 extrapoliert

The four mean craniofacial shapes of the malocclusion groups differed mainly in mandibular shape and size (relative to other facial structures) as well as in the orientation and position of the teeth (Figs. 2, 3; Table 2). The average mandible was more prognathic in the class III group and retrognathic in the class II group. Compared to class III individuals, the class II group had a more flexed gonial angle and a shorter chin. In the anterior open bite group the mandible was short, the gonial angle obtuse, and the maxilla and mandible showed the greatest divergence.

**Fig. 4:**
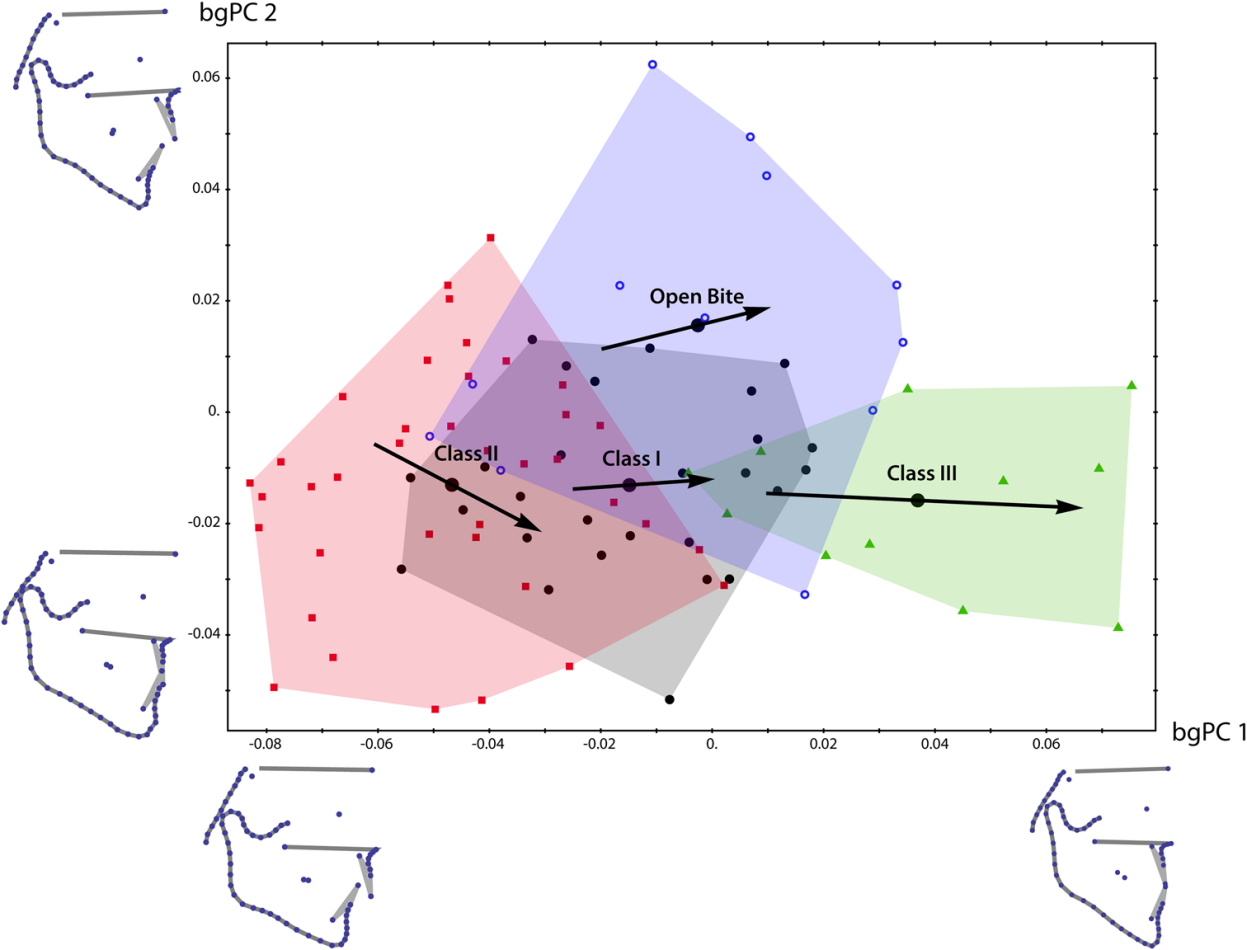
Scatter plot of the first two between-group principal components (*bgPCs*) of shape. Class I patients are shown by *black filled circles*, class II patients by *red squares*, class III patients by *green triangles*, and open bite patients by *blue open circles*. *Large black circles* represent group averages. *Black arrows* depict linear estimates of average growth in the four groups (linear regressions of shape on age). *Arrows* are scaled to correspond to the average shape change from 8- to 20-year-old individuals in each group; the length of the arrow is an estimate of the magnitude of overall shape change in this age period, whereas differences in direction indicate differences in the pattern of shape change. Shape differences associated with PC axes are visualized by shapes corresponding to scores of −0.8 and 0.8 for PC1 (*anteroposterior* direction) and −0.6 and 0.6 for PC2 (*vertical* direction). (Units in Procrustes distance) **Abb. 4** Scatter-Plot der ersten 2 “between-group principal components” (*bgPCs*) der Form. *Schwarze geschlossene Kreise* Klasse-I-Patienten, *rote Vierecke* Klasse-II-Patienten, *grüne Dreiecke* Klasse-III-Patienten, *blaue offene Kreise* Patienten mit offenem Biss. *Große schwarze Kreise* stehen für Durchschnittswerte in den Gruppen, *schwarze Pfeile* für lineare Schätzungen des durchschnittlichen Wachstums in den 4 Gruppen (lineare Regressionen: Form—Alter). Skalierungen auf den Pfeilen entsprechen den durchschnittlichen Formveränderungen zwischen 8 und 20 Lebensjahren in jeder Gruppe, die Länge des Pfeils ist eine Abschätzung der Formveränderungen insgesamt in diesem Altersintervall, unterschiedliche Richtungen dagegen zeigen Unterschiede im Muster der Formveränderung an. Mit den PC-Achsen assoziierte Formunterschiede sind visualisiert durch Formen entsprechend Scores von −0,8 und 0,8 für PC1 (anteroposteriore Richtung) sowie −0.6 und 0,6 für PC2 (vertikale Richtung). (Einheiten in Prokrustes-Distanzen)

The first two between-groups PCs in Fig. 4 accounted for 42% of total shape variation between the patients and for 97% of shape variation between the four group means. The shape differences among the three Angle classes were closely aligned with bgPC 1 (anterior–posterior direction), where the class I group was between class II and class III individuals. The class II and open bite groups were considerably more variable along bgPC 2 (vertical direction) than the other groups (Fig. 4).

In Fig. 4, the bgPCA shows substantial individual overlap between the groups. Despite this overlap, the groups without open bite differed significantly in mean shape (*p* < 0.001 for all pairwise tests after Bonferroni correction). Individuals with open bite differed significantly from the class II group only (*p* < 0.001 after Bonferroni correction).

The black arrows in the bgPCA plot (Fig. 4) represent the average shape difference between 8-year-old patients and 20-year-old patients, derived from the linear regressions of shape on age. The arrows head in similar directions, indicating that growth patterns were similar across all four groups and that the shape differences between the four groups were already present in childhood. Hence, the variation of age within each group did not obscure the presented group mean differences.

**Fig. 5:**
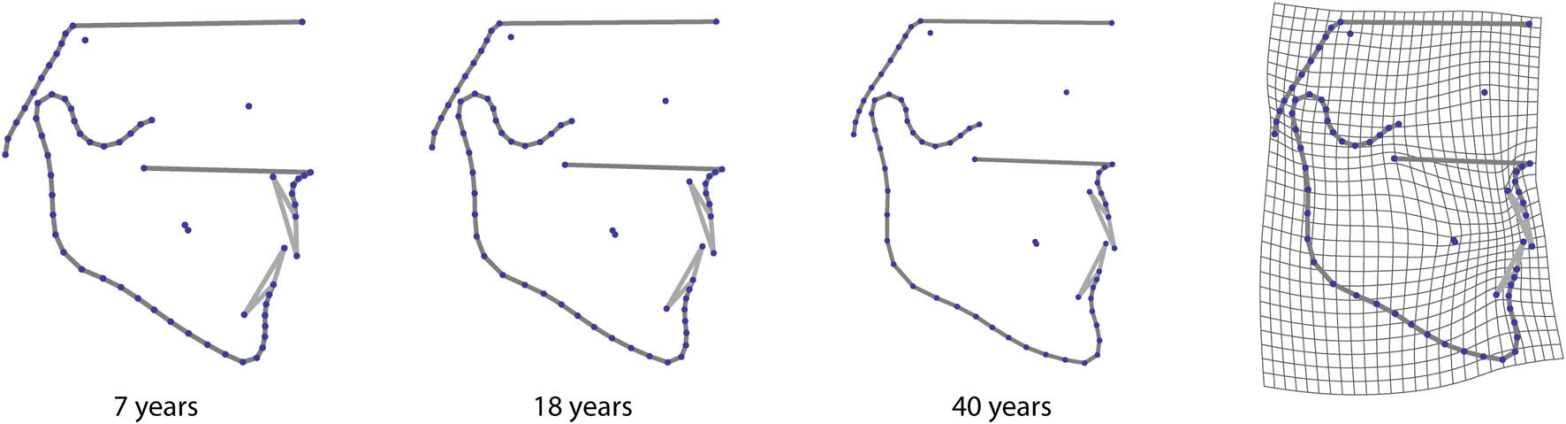
Visualization of the average age-related shape changes, pooled over all four groups. The first three illustrations show the average shapes of 7-, 18-, and 40-year-old patients, estimated from a linear regression of shape on age. The 40-year shape is an extrapolation of the actual growth pattern. The deformation grid illustrates shape differences between the 7- and 40-year shapes by threefold extrapolation of the actually observed growth **Abb. 5** Visualisierung der durchschnittlichen altersbezogenen Formveränderungen, gepoolt für alle 4 Gruppen. Die ersten 3 Bilder zeigen die durchschnittlichen Formen bei 7, 18 und 40 Jahre alten Patienten, geschätzt entsprechend einer linearen Form-Alter-Regression. Die Form bei 40 Jahren ist eine Extrapolation des tatsächlichen Wachstumsmusters. Das Deformationsgitter stellt die Unterschiede zwischen den 7- und 40-Jahre-Formen durch dreifache Extrapolation des beobachteten Wachstums dar

In Fig. 5, the average shape change within the observed growth period, pooled over all diagnostic groups and genders, is shown. With increasing age, the mandibular ramus increased in height and the gonial angle flexed, the chin projected forward, and the lower incisors were displaced anteriorly relative to the upper incisors.

In Figs. 2, 3 and 4, it can be seen that bony structures differed across the groups even though the diagnostic criteria referred to the first molar interdigitation exclusively. When using landmarks located only on bony structures, 64.5% of the patients were classified correctly with regard to their occlusion in the discriminant function analysis. Using molar and incisor landmarks, 80.3% of the patients were classified in agreement with the original assignment of occlusion.

**Fig. 6:**
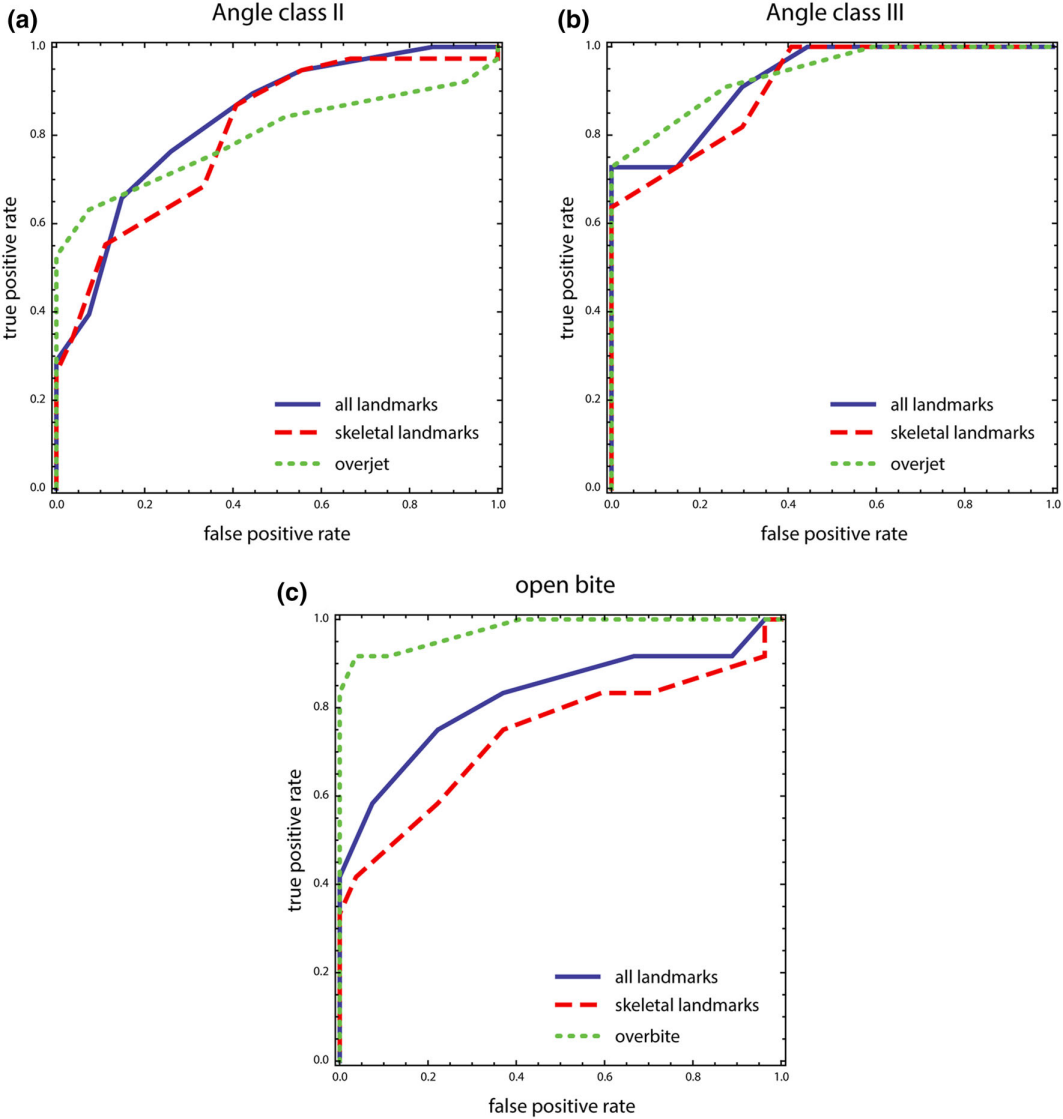
Receiver operating characteristic (ROC) curves for **a** Angle class I versus Angle class II, **b** Angle class I versus Angle class III, and **c** Angle class I versus open bite. Classifications in **a** and **b** are based on bgPC 1 using all landmarks (*solid blue lines*) and skeletal landmarks only (*dashed red lines*). *Dotted green lines* denote classifications based on overjet. In **c**, *blue* and *red lines* are computed from bgPC 2 using all landmarks and skeletal landmarks, respectively (*green line* refers to overbite) **Abb. 6** ROC (“receiver operating characteristic”)-Kurven für **a** Angle-Klasse I vs. Klasse II, **b** Angle-Klasse I vs. Klasse III und **c** Angle-Klasse I vs. offener Biss. Die Klassifikationen zu **a** und **b** basieren auf bgPC 1 unter Verwendung aller Landmarken (*durchgezogene blaue Linie*) bzw. der skelettalen Landmarken (*gestrichelte rote Linie*). *Gepunktete grüne Linien* zeigen auf dem Overjet basierende Klassifikationen. **c** *Blaue* und *rote Linien* wurden auf der Basis von bgPC 2 unter Verwendung aller bzw. nur der skelettalen Landmarken berechnet (die *grüne Linie* basiert auf dem Overbite)

Figure 6 shows the ROC curves computed separately for three different datasets: a dataset including all landmarks, another containing skeletal landmarks only, and a third one for standard linear measurements of incisor overjet and overbite. The ROC curves for Angle class I versus class II groups (Fig. 6a) and group I versus group III (Fig. 6b) yielded three similar curves, indicating that the three datasets have a similar power to discriminate between the malocclusion groups. Table 3 lists the values for the areas under the ROC curves, which ranged from 0.74 to 0.98.

**Tab. 3:** Areas under the receiver operating characteristic (*ROC*) curves. Areas represent the sensitivity of the tested parameters. A perfect test without false positives and false negatives yields 1 **Tab. 3** Flächen unter den ROC (“receiver operating characteristic”)-Kurven. Die Flächen repräsentieren die Sensitivität der untersuchten Parameter. Ein perfekter Test ohne falsch-positive bzw. falsch-negative Ergebnisse ergibt 1

	All landmarks	Skeletal landmarks	Overjet	Overbite
Class I vs. class II	0.84	0.80	0.80	–
Class I vs. class III	0.93	0.87	0.94	–
Class I vs. openbite	0.82	0.74	–	0.98

The Angle class III group was generally more distinguishable from the class I group than the class II sample from the class I sample. In Fig. 4, the larger overlap between groups I and II than between groups I and III corroborates this statement. The comparisons between the class I and open bite group is illustrated in Fig. 6c. Here the incisor overbite discriminated the groups to a higher degree than other landmark datasets.

## Discussion

Deviant size, shape, and inclination of the maxillae contribute to craniofacial phenotype variation and malocclusion [11, 30]. Using GM and bgPCAs, we found a significant association between craniofacial shape and malocclusion. The average shape of the mandible differed across the four groups and presented a more acute gonial angle as well as a sagittally and vertically shorter lower face in the distocclusion group, while the mesiocclusion group showed the opposite pattern.

In the open bite group, both the maxilla and the mandibular body differed in their average inclination from the neutrocclusion group. The maxilla was inclined upward and the mandible downward. The downward rotation of the mandibular body was not primarily located at the gonion but along the entire posterior half of the mandibular corpus. This finding displays a similarity with Björk’s mandibular backward rotation around the last occluding molars in growing individuals [4].

In contrast to maxillary and mandibular shapes, the cranial base and upper face were similar across the groups as illustrated by Figs. 2 and 3. These structures suggested less association with malocclusion. The class I and class II groups showed almost identical dimensions for the average distance between sella and nasion while the mesiocclusion and open bite groups were also alike in their average sella-nasion measurements, but shorter (Table 2). This resemblance of the mesiocclusion and open bite groups is in agreement with Ellis and McNamara [9], who reported similar cranial base morphology for class III individuals with and without open bite.

Angulation and size of the cranial base have been considered causative factors in developing class II or class III relationships [10, 14, 22]. In our study the mesiocclusion group showed, on average, the greatest cranial base angle (nasion-sella-basion), the neutrocclusion group the smallest angle, and the means of the class II and open bite groups were between these. However, in accordance with recent investigations [2, 32], the differences in cranial base angle were small (ranging from 128.5° to 132.3°; Table 2) and they were not statistically significant, questioning the relevance of cranial base flexion for malocclusion.

In addition to skeletal differences, the malocclusion groups differed in incisor position. Proclined maxillary incisors and retroclined mandibular incisors in the class III group as well as protruded mandibular incisors in the class II group indicate dentoalveolar compensation for functional interarch relations under varying jaw relationships [26, 37, 38].

Because our sample was of mixed age, we observed growth effects on craniofacial shape from age 8 years onward. At this age the median cranial base, the presphenoid, and the cribriform plate as well as the cranial base angle are stable [1, 3, 22]. In the observed growth period, the mandibular ramus increased in height while the gonial angle decreased and the chin projected forward (Fig. 5). In the bgPCA plot in Fig. 4, the growth effects are represented as arrows from the average 8-year-old shape to the average 20-year-old shape within each group. The Angle class I group had the shortest arrow, i.e., the least alteration in shape between 8 and 20 years, while the other three groups showed more growth changes. The class III patients experienced the greatest extent of shape change. This unique divergence from the growth of the class I group may represent unbalanced growth rates or remodelling of craniofacial structures in class III individuals. The similarities of class I and class II craniofacial growth patterns, leading to a flattening of the face, agree with Yoon and Chung [40].

Despite the clear differences in average shape across the four groups, the individual distribution of craniofacial shape overlapped between these groups (Fig. 4). The deformation grids of Fig. 3 explicitly visualise the sites, where between-group differences of the phenotypes exist. Thus, craniofacial shape is not the sole cause of malocclusion but it clearly contributes to dental misalignment. The mesiocclusion group overlapped least with the neutrocclusion group, as compared to class II and open bite individuals. Craniofacial morphology seems to play a more important role for class III malocclusion than for other malocclusions.

The mesiocclusion group was also less variable along the vertical dimension (bgPC 2) than the other groups. Unique proportions and minimum skeletal variability explain this characteristic of the mesiocclusion group. The predominantly hyperdivergent but horizontally short maxilla of the class III group showed increased maxillary and mandibular molar dentoalveolar heights (Fig. 3) and yielded high mean values but small standard deviations for the angles SN–PP and PP–MP (Table 2).

The overlap of craniofacial shape among different types of occlusion is also reflected by the imperfect classification of patients based on the skeletal measurement points (Fig. 6; Table 3). The discriminant function analysis is powerful in determining whether a set of landmarks is effective in predicting inclusion to a category. Using skeletal landmarks and semilandmarks exclusively, classification was most successful for the class III group, again indicating its distinct craniofacial shape. However, malocclusion could also not be predicted perfectly when using molar and incisor landmarks only, presumably because of averaging bilateral structures on the tracing. About 20% of patients were classified incorrectly (Table 3).

Two-dimensional cephalograms apparently do not allow for a correct diagnosis in every instance. The actual occlusion was diagnosed according to the molar relationship on plaster casts. If diagnosis of malocclusion has to be made on the basis of cephalograms alone, the ROC statistics show that the geometric morphometric quantification of craniofacial shape does not outperform simple linear measurements of horizontal or vertical projections of the distance between the incisal edges (overbite, overjet). For the diagnosis of open bite, a measure of overbite is diagnostically even more effective than the multivariate approach.

As mentioned above, the malocclusion groups overlapped in shape space. Moreover there were no discrete clusters within the malocclusion groups. Moreno Uribe et al. [27, 28] studied craniofacial variation in distocclusion and mesiocclusion patients using numerous conventional cephalometric measurements, which were ranked by PCA, and reported the presence of multiple clusters of patients with respect to their craniofacial phenotypes. However, these “clusters” strongly overlapped even in a canonical variate analysis and, hence, are not clusters in the sense of separated groups of individuals. Neither their results nor our data support a distinctive and unique typology of craniofacial shape and occlusal pattern. Corroborating our findings, a morphometric study of adult skeletal open bite [19] also reported substantial variation in sagittal and vertical directions.

Limitations of our study include sample size, distribution of gender within groups, restriction to 2D data, and a cross-sectional retrospective study type. Within these limitations, the geometric morphometric analysis allowed for novel insights into craniofacial shape variation within and between the neutrocclusion and malocclusion groups.

## Conclusions

Shape and relative position of the mandible contributed to malocclusion but varied considerably within the malocclusion groups. The maxillary shape and the cranial base showed less variation.

Skeletal morphology plays a larger role in individuals with class III malocclusion than in other malocclusions. Class III patients were considerably less variable in vertical direction than the other groups. The Angle class I group showed the least alteration in shape between 8 and 20 years.

The anterior open bite group had the most hyperdivergent skeletal pattern but was highly variable. The open bite group overlapped in its distribution with all three other groups and did not represent its own entity.

Geometric morphometrics proved to be a powerful research tool. Nonetheless, for the pure purpose of diagnosing malocclusion in an individual, standard cephalometric measurements as well as overjet and overbite appear to be equally or more efficient than geometric morphometric descriptors of dentofacial shape.
